# Expression Changes in the Stroma of Prostate Cancer Predict Subsequent Relapse

**DOI:** 10.1371/journal.pone.0041371

**Published:** 2012-08-01

**Authors:** Zhenyu Jia, Farah B. Rahmatpanah, Xin Chen, Waldemar Lernhardt, Yipeng Wang, Xiao-Qin Xia, Anne Sawyers, Manuel Sutton, Michael McClelland, Dan Mercola

**Affiliations:** 1 Department of Pathology and Laboratory Medicine, University of California Irvine, Irvine, California, United States of America; 2 Proveri, San Diego, California, United States of America; 3 AltheaDx, San Diego, California, United States of America; 4 Department of Bioinformatics, Institute of Hydrobiology, Chinese Academy of Sciences, Wuhan, Hubei, China; 5 Vaccine Research Institute of San Diego, San Diego, California, United States of America; Baylor College of Medicine, United States of America

## Abstract

Biomarkers are needed to address overtreatment that occurs for the majority of prostate cancer patients that would not die of the disease but receive radical treatment. A possible barrier to biomarker discovery may be the polyclonal/multifocal nature of prostate tumors as well as cell-type heterogeneity between patient samples. Tumor-adjacent stroma (tumor microenvironment) is less affected by genetic alteration and might therefore yield more consistent biomarkers in response to tumor aggressiveness. To this end we compared Affymetrix gene expression profiles in stroma near tumor and identified a set of 115 probe sets for which the expression levels were significantly correlated with time-to-relapse. We also compared patients that chemically relapsed shortly after prostatectomy (<1 year), and patients that did not relapse in the first four years after prostatectomy. We identified 131 differentially expressed microarray probe sets between these two categories. 19 probe sets (15 genes overlapped between the two gene lists with *p*<0.0001). We developed a PAM-based classifier by training on samples containing stroma near tumor: 9 rapid relapse patient samples and 9 indolent patient samples. We then tested the classifier on 47 different samples, containing 90% or more stroma. The classifier predicted the risk status of patients with an average accuracy of 87%. This is the first general tumor microenvironment-based prognostic classifier. These results indicate that the prostate cancer microenvironment exhibits reproducible changes useful for predicting outcomes for patients.

## Introduction

Prostate cancer is the most frequently diagnosed male cancer and the second leading cause of cancer death in men in the United States [1]. Each year in the US, there are approximately 230,000 new cases of prostate cancer and approximately 195,000 radical prostatectomies are performed [2]. However, few patients may be saved by these treatments because only a minority of cases will die of the disease if left untreated. The number needed to treat to save one life estimated in two studies was 12–15 [3] and up to 48 [4]. Numerous nomograms and related prediction methods have been created based on clinical variables at the time of diagnosis but, to date, such tools have provided limited advice regarding which patients harbor aggressive disease requiring radical treatment possibly followed by adjuvant therapy and which patients may be suitable for a more conservative active surveillance program [5]–[9].

Enormous efforts have been invested in the development of biomarkers for prognosis of prostate cancer with an emphasis on features of the tumor epithelial component in retrospective samples. However, few accepted and clinically employed biomarkers have been developed. One barrier to biomarker discovery may be the cell-type heterogeneity and the polyclonal/multifocal nature of the accumulated genetic alterations at the time of diagnosis [10]–[13]. In contrast, the tumor microenvironment exhibits much more limited mutations and loss of heterozygosity (LOH) [14] but may respond to paracrine signals from nearby tumor. It has been shown that the microenvironment of selected cases exhibit distinct histological changes termed “reactive stroma” with distinct expression profiles which correlate with poor outcome [15], [16]. Indeed, we have demonstrated that tumor-associated stroma without regard to subtype possesses unique expression profiles when compared to normal stroma. We used these gene expression changes to develop a classifier that can accurately diagnose the presence of tumor in prostate cancer cases even if the samples used for analysis do not contain recognizable tumor [13]. This approach has clinical potential for resolving hundreds of thousands of ambiguous biopsies performed in the US every year, which will greatly improve disease management and save lives. Similarly, useful diagnostic information has been obtained from examining the methylation status of GSTP1 and APC genes in negative initial prostate biopsies [17]. The differential expression and epigenetic profiles in tumor-associated stroma compared to the normal stroma may reflect stroma responses to tumor paracrine factors as well as other influences. If the quality and quantity of such responses correlate with clinical outcome such as the indolent or aggressive phenotypes, then the stroma response to nearby tumor might be useful for deriving a general rule for prognosis. Other researchers have observed such differences in breast cancer [18]. In this study, we tested this hypothesis by comparing gene expression profiles between stroma samples among patients with known different outcomes, regardless of histology, and identifying 115 probe sets for which the expression levels are significantly correlated with times-to-relapse. We also compared expression profiles between a subset of stroma samples from patients that relapsed quickly and stroma samples from patients that had not relapsed after more than four years. We identified 131 probe sets that had altered expressions. There were 19 probe sets (15 unique genes) in common between these two gene lists. We then derived a 15-gene classifier. The overall accuracy was 87% when the classifier was tested on 47 independent test samples. Pathway analysis and Gene Ontology studies indicated these 15 genes are significantly enriched for genes that are involved in apoptosis-related processes. These studies supported the possibility that stroma is a practical basis of risk assessment.

## Materials and Methods

### Prostate Cancer Patient Samples and Expression Analysis

Our data sets GSE8218 and GSE17951, which are publically available in the Gene Expression Omnibus (GEO) database, are based on post-prostatectomy frozen tissue samples obtained by informed consent using IRB-approved and HIPPA-compliant protocols. All tissues were collected at surgery and escorted to pathology for expedited review, dissection, and snap freezing in liquid nitrogen. Clinical follow-up data was assimilated by the UCI SPECS program and maintained in a relational database. RNA for expression analysis was made directly from frozen tissue following dissection of OCT (optimum cutting temperature compound) blocks prepared from the snap frozen samples with the aid of a cryostat. Stroma from tumor-bearing samples was prepared from the OCT-embedded tissue that was mounted in a cryostat by etching a line between tumor and stroma with a scalpel and then preparing frozen sections which appear as two pieces one of which is tumor adjacent stroma as described in [13]. Before perfection of this method, some stroma was prepared by hand dissection of frozen tissue with a scalpel. In order to avoid contamination, the hand method required leaving a gap between tumor and stroma of 0.5–1.0 mm and the resulting stroma is termed “near” stroma.

For expression analysis 50 micrograms (10 micrograms for biopsy tissue) of total RNA samples were processed for hybridization to Affymetrix GeneChips (GSE17951: U133 Plus 2.0 platform; GSE8218: U133A platform). For these two data sets, the distributions for the four principal cell types [tumor epithelial cells, stroma cells, epithelial cells of benign prostatic hyperplasia (BPH), and epithelial cells of dilated cystic glands] were estimated by up to four pathologists, whose estimates were averaged as described [19], [20].

Data set GSE25136 (U133A platform), which consists of 79 tumor-bearing cases (>10% tumor cells), was independently developed and used as a test set. The cell-type distribution of this data set was estimated using CellPred, an *in silico* method to determine the tumor percentage of samples based on the expression values for the multi-gene signatures that are invariant with tumor surgical pathology parameters of Gleason and stage (available at http://www.webarraydb.org/webarray/index.html) [20]. Note that the cell-type distribution of data sets GSE8218 and GSE17951 were provided by up to 4 pathologists [19], whereas the cell-type distribution of data set GSE25136 was estimated by *in silico* method [20].

### Statistical Methods

Normalization was carried out across multiple data sets using the ∼22,000 probe sets in common to all Data sets. First, data set GSE8218 was quantile-normalized using the function ‘normalizeQuantiles’ of the LIMMA routine [21]. Data sets GSE17951 and GSE25136 were then quantile-normalized by referencing the normalized data set GSE8218 using a modified function ‘REFnormalizeQuantiles’ which is available at the SPECS website (http://www.pathology.uci.edu/faculty/mercola/UCISpecsHome.html) [22]. The LIMMA package from Bioconductor was used to detect differentially expressed genes. Prediction Analysis for Microarrays (PAM [23]), implemented in R, was used to develop an expression-based classifier from the training sets and then applied to the test sets without further change.

## Results

### Gene Expression Associated with Risk

Two methods were employed to define genes differentially expressed in stroma of high and low risk cases. Short disease-free survival (DFS) time is a commonly used indicator of aggressiveness [24]–[26]. First, we defined aggressive prostate cancer cases as those patients who experienced disease relapse within 1 year after prostatectomy, and indolent (or less aggressive) cases as those patients who either relapsed later than 4 years after surgery or who did not relapse and had at least 4 years’ follow-up data available. Based on these criteria, we identified 40 rapid relapse patient samples containing pure stroma that were near to tumor and 9 patient samples with indolent disease containing pure stroma that were near to tumor from data set GSE8218. Of these arrays we randomly selected 8 rapid relapse patient samples and 7 indolent patient samples as the training sets and compared the expression profiles of these two groups using LIMMA. Genes with *p* values <0.05 and fold change >1.6 (either up-regulated or down-regulated) were identified and used to develop a PAM classifier. The resulting classifier was subsequently tested against the patient samples that had not been used for training (32 rapid relapse patient samples and 2 indolent patient samples). This process was repeated 1,000 times and 3,625 probe sets were selected at least once out of 1,000 times based on criterions of *p* values <0.05 and fold change >1.6. The average sensitivity and specificity of the cross-validation process were 69% and 82%, respectively. A total of 131 probe sets were selected by PAM no less than 500 times out of the 1,000 iterations.

Second, in order to identify probe sets associated with a broader class of risk values, the data set GSE8218 again was used to identify probe sets that correlate with disease-free survival time, including patients that relapsed between one and four years after surgery. Data set GSE8218 included 49 pure stroma samples from 49 patients who underwent prostate cancer relapse after surgery. Note that the 49 stroma samples used for correlation analysis are not identical to the 49 stroma cases used for rapid relapse *vs.* indolent comparison which consist of 44 relapsed cases (in common with the cases used for correlation analysis) and 5 non-relapsed cases. We analyzed the 49 stroma samples from relapse case by a correlation analysis and identified 115 DFS-associated probe sets using Pearson’s correlation analysis with correlation coefficients >0.46 and associated *p* values <0.001). The Pearson’s correlation coefficients for these 115 probe sets range from −0.46 to −0.61 or from 0.46 to 0.69. Different disease-progression relevant genes, beyond those found in early and late relapse cases (131 genes above) were assumed to be uncovered in this gene identification step because median-risk cases (relapse time between 1 year and 4 years) were included.

There were 19 common probe sets between the 131 probe sets identified by permutated PAM analysis and the 115 probe sets identified from correlation analysis. A simulation study showed that the chance of observing 19 overlap between randomly selected 131 probe sets and 115 probe sets from a basis of 22,000 probe sets is <0.0001. Thus these 19 overlapping probe sets (a figure greater than random) represent significant agreement between two non-identical sets of case using different methods of analysis. The 19 common probe sets, which represent 15 unique genes, are listed in **Table 1**. Example plots of expression of these probe sets *vs.* the DFS time are shown in **Figure S1**.

**Table 1:** 19 probe sets that are consistently associated with relapse.

Probe Set ID	Gene Title	Gene Symbol	Fold change
**207574_s_at**	**growth arrest and DNA-damage-inducible, beta**	**GADD45B**	**4.86**
**209304_x_at**	**growth arrest and DNA-damage-inducible, beta**	**GADD45B**	**4.53**
***213757_at***	***Transcribed locus, weakly similar to XP_001478155.1 PREDICTED: hypothetical protein [Mus musculus]***	***–-***	**3.70**
***202284_s_at***	***cyclin-dependent kinase inhibitor 1A (p21, Cip1)***	***CDKN1A***	***2.65***
***218380_at***	***NLR family, pyrin domain containing 1***	***NLRP1***	***1.57***
***202454_s_at***	***v-erb-b2 erythroblastic leukemia viral oncogene homolog 3 (avian)***	***ERBB3***	***0.55***
205776_at	flavin containing monooxygenase 5	FMO5	0.55
212314_at	KIAA0746 protein///serine incorporator 2	KIAA0746///SERINC2	0.43
202203_s_at	autocrine motility factor receptor	AMFR	0.41
211478_s_at	dipeptidyl-peptidase 4 (CD26, adenosine deaminase complexing protein 2)	DPP4	0.40
203716_s_at	dipeptidyl-peptidase 4 (CD26, adenosine deaminase complexing protein 2)	DPP4	0.39
205261_at	progastricsin (pepsinogen C)	PGC	0.38
***210317_s_at***	***tyrosine 3-monooxygenase/tryptophan 5-monooxygenase activation protein, epsilon polypeptide***	***YWHAE***	***0.34***
219850_s_at	ets homologous factor	EHF	0.33
203400_s_at	transferrin	TF	0.33
***202687_s_at***	***tumor necrosis factor (ligand) superfamily, member 10***	***TNFSF10***	***0.31***
***201123_s_at***	***eukaryotic translation initiation factor 5A***	***EIF5A***	***0.15***
217566_s_at	transglutaminase 4 (prostate)	TGM4	0.03
206260_at	transglutaminase 4 (prostate)	TGM4	0.01

The entries in boldface are genes associated with apoptosis while the *italicized* entries are genes associated with cell death.

Classifier development and testing with independent data sets.

The 19 overlapping probe sets were used to develop a classifier. From 40 rapid relapse cancer samples from the first step containing stroma near tumor, we selected 9 samples with the shortest DFS times, which were combined with all 9 samples containing stroma near indolent tumor to form a training set. We used the 19 probe sets identified in the previous step as PAM [23] input to develop a classifier based on these 18 training samples. The observed status of the training cases as aggressive case or indolent case was specified. All 19 probe sets were retained by the PAM optimizing process with a final training accuracy of 88.9% (**Table 2**).

**Table 2:** Performance of the 15-gene classifier versus random classifiers.

	Accu. (%)	Sens. (%)	Spec. (%)
***Training***			
9 HRs stroma samples *vs* 9 LRs stroma samples^1^	88.9/70.3	88.9/68.7	88.9/71.9
***Test***			
31 HRs stroma^1^	87.1/50.9	87.1/50.9	–
2 HRs with <10% tumor^1^	100/45.4	100/45.4	–
3 LRs with <10% tumor^1^	100/44.1	–	100/44.1
7 HRs stroma^2^	85.7/23.3	85.7/23.3	–
2 HRs with <10% tumor^2^	100/45.4	100/45.4	–
2 LRs with <10% tumor^2^	50/74.4	–	50/74.4

1 GSE8218, Affymetrix U133A

2 GSE17951, Affymetrix U133Plus 2. 2. The first/second value in each cell represents the result from 15-gene classifier/the average of 1000 random classifiers.

A heat map (**Figure S2**) illustrates that the 194 genes (the combination of the 131 probe sets identified by PAM analysis and the 115 probe sets identified from correlation analysis) had distinct profiles between rapid relapse cases and indolent cases in the 18 training stroma samples. A volcano plot (**Figure S3**) illustrates that some of the probe sets have large fold changes and low p values. The 115 probe sets have 19 probe sets (15 unique genes) in common with the 131 probe sets identified from the permutated PAM analysis. The volcano plot in **Figure S3** illustrates that these 19 probe sets are among the most promising probe sets which have the largest fold changes and lowest p values.

In order to provide an objective test of the prognostic classifier, 47 independent test samples including 36 samples from data set GSE8218 and 11 samples from data set GSE17951 (not used in training) were employed for testing. A sensitivity of 88.1% and a specificity of 80% were observed yielding the average accuracy was 87% (Table 2, ***Test***). The overall positive predictive value (PPV) and the negative predictive value (NPV) of the test based on the 47 independent samples were 97.9% and 44.4%, respectively.

**Figure 1:**
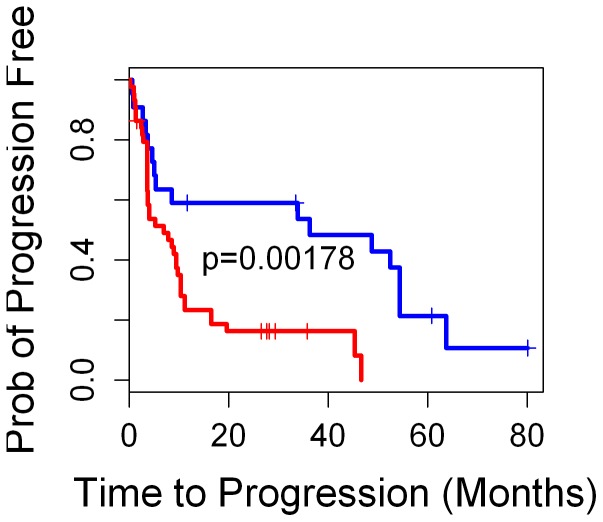
Kaplan-Meier analysis of 66 test samples based on the prediction made by the 15-gene prognostic classifier.

In order to test further whether the 15-gene prognostic classifier generally applies to entire range of outcomes and is not limited to the specific selected survivorship selected for training in the first step, we tested 19 samples (not included in training) from patients who either suffered relapse between year 1 and year 4 after surgery or did not relapse but had less than 4 years’ follow-up data. These 19 samples included 9 stroma samples from near tumor and 10 tumor-bearing samples (tumor <10%). The Kaplan-Meier analysis indicated that the 15-gene prognostic classifier dichotomized these ambiguous samples into two groups with significantly distinct risks (*p* = 0.02). These observations indicated that the combination of a training method based on selected survivorship in combination with a correlation method that utilized the full available range of disease-free survival times yielded a classifier with accurate results when applied to an independent test cohort. A Kaplan-Meier representation of the test results for the 47 test samples in combination with the test results for these 19 median-risk stroma samples is summarized in **Figure 1**. These results yielded a probability of chance separation of the predicted classifications with a *p* = 0.0018.

To measure the significance of the 19 probe sets classifier, we did an experiment based on sets of genes selected at random. We randomly selected 19 probe sets from among all 22,283 probe sets and reran the training and test, as described above. This random process was repeated 1,000 times. The averages of the operating characteristics are given in **Table 2**. Only 7% of the 1000 random classifiers had equal or better performance than the prognostic classifier.

We also checked if including clinical information, such as Gleason Scores, tumor stage and pre-operative PSA added prognostic value to the classifier. We used these three variables in combination with the 19 probe sets as PAM input and let PAM select the best predictive features. None of these three variables were picked by PAM. In addition, we analyzed 65 cases (18 training cases and 47 test cases in Table 2) using a multivariate Cox proportional hazards regression, where age, Gleason sum, TNM and pre-op PSA are compared to the prediction made by our classifier. Only classifier prediction (*p* = 0.0005) and TNM (*p* = 0.0383) were significantly associated with survival. The result indicated that gene signature has better predictive value and adds predictive value to known clinical and pathological variables.

### Test on Stroma Samples Far from Tumor and Tumor Samples with Low Amounts of Stroma

The 19 probe sets (15 genes) form a prognostic signature specific to stroma near tumor. To examine whether the 15-gene classifier extended to stroma that is far from primary tumor, we tested the classifier on 9 indolent stroma samples 8 of which are *far* from the primary tumor, taken from a zone contralateral to the tumor site. The accuracy or specificity was only 11.1% (data not shown). Thus, when stroma is tested from remote positions with a low likelihood of being affected by tumor paracrine factors, a stroma response represented by the expression changes of these 15 genes was not detected in contrast to such changes detectable in stroma near tumor. To check whether the 15-gene classifier was insensitive to large amounts of contaminating tumor, we tested it on 117 tumor-bearing samples (>10% tumor cells with average of 48.5% of tumor component) in three data sets (GSE8218, GSE17951, and GSE25136) with an overall accuracy of 41%. However, when the classifier was tested on 9 samples that contain <10% tumor cells, the accuracy was 89% (**Table 2**). Thus for the intended clinical use of the assay, it will be important to sample stroma that is adjacent to but free of tumor cells.

### Function Analysis for the Classifier Genes

We analyzed the 19 probe sets (15 genes) using the DAVID bioinformatics tool [27]. The 15 genes are significantly enriched in genes associated with apoptosis and with cell death (p<0.001 and Benjamin score <0.05) (**Table 1**, in boldface and/or italicized). We further analyzed the 194 genes (the combination of the 131 probe sets identified by PAM analysis and the 115 probe sets identified from correlation analysis) using a pathway analysis tool from MetaCore (GeneGo Inc.). The filtering system of MetaCore helped limit our search to those genes that have been reported in specific tissue, for example, prostate tissue. The filtered genes were used to build the signaling pathways. The statistically significant pathways had to meet the FDR <0.05 and multiple genes (>2) significantly associated with the biological pathways. To analyze the 194 genes, we used ‘smooth muscle + disease biomarker’ and ‘prostatic neoplasms transcription’ as filtering parameters. The results of MetaCore pathway analysis are listed in **Table S1**.

## Discussion

We previously showed that there are hundreds of significant gene expression changes between tumor-adjacent stroma and normal stroma that were used to develop a stroma-specific high accuracy Diagnostic Classifier for detecting the presence-of-tumor based on the RNA expression of stroma alone [13]. These stroma-specific expression changes are likely to be due to the reaction of stroma to the tumor-derived paracrine mediators as well as a possible “field effect”. Here we further hypothesized that there may be expression differences between the stroma of indolent and aggressive tumors, which could be utilized for clinical prognosis. In order to test this hypothesis, we compared gene expression profiles between tumor-adjacent stroma samples from patients that experienced rapid relapse and tumor-adjacent stroma samples from patients that did not experience relapse or for which relapse took many years. 40 stroma samples from rapid relapse and 9 stroma samples from indolent cases were subjected to a permutation process to identify differentially expressed genes. In each of 1,000 iterations/resample, we used 31% of the stroma samples (8 out 40 rapid relapse stroma samples and 7 out of 9 indolent stroma samples) for training and used the remaining stroma samples for testing. Owing to the fact that we had small number of samples for training, we selected small but similar numbers (8 and 7) for each iteration in order to give room for resampling (permuted analysis). The advantages for this scheme are three fold. First, it was a balanced analysis in each resample. Second, such scheme is robust to potential ‘bad’ samples since bad samples may be excluded in many resample combinations. Third, such scheme can dramatically increase the detection base (a total of 3625 probes were identified by 1,000 resamples). However, we only selected 131 probe sets that were identified more than 500 times in the 1,000 iterations to reduce the chance of false identifications. We also identified 115 probe sets of which the expression levels in tumor-adjacent stroma are significantly correlated with the disease-free survival times of the patients who underwent disease relapse. The 19 common probe sets (15 unique genes) of these two significant gene lists were used to develop a PAM-based classifier, which had an average accuracy of 87% when it was tested on 47 independent tumor-adjacent stroma samples.

Recently, it has been reported that in breast cancer any set of 100 genes or more selected at random has a 90% chance to be significantly associated with outcome, and most published signatures are not significantly more associated with outcome than random predictors [28]. In order to address this problem, we generated random classifiers based on the *same* training samples and the 1,000 sets of 19 probe sets selected at random and tested these random classifiers with the *same* test samples as used for testing the 19-probe set Prognostic Stroma Classifier. The average number of probe sets selected by PAM in the 1,000 random sets is 3.7 which are assumed to be a noise. That is for any randomly picked set of 19 probe sets, a small number of probe sets would be correlating with the high/low risk status by coincidence, which explains why the average training accuracy of random classifiers was ∼70%. However, these random classifiers would not work for independent test sets. On the contrary, the 19 probe sets were identified through both rigorous approaches; therefore, they are potentially general prognostic markers that apply to other test sets. The comparison favored our 15-gene (19 probe set) classifier over those classifiers generated through random processes (**Table 2**).

A number of genes identified here for classifier development have been observed in other studies of RNA expression in the stroma of prostate tissue. We compared the total of 227 probe sets or 194 unique genes identified here with stroma-specific probe sets previously identified in three studies as useful for diagnosis. There are 2 genes in common (PROM1, GPM6B) with the 339 probe sets used to develop our diagnostic classifier [13]; 3 genes (SEL1L3, KRT19, and KRT7) in common with the 119 genes differentially expressed gene of Joesting *et al.* [29], and 3 genes (NKX3-1, TPD52, and GALNT3) in common with the 44 genes that were differentially expressed between tumor-associated stroma and nontumor stroma from 5 patients [30]. These observations indicate that the prognostic signatures in stroma are largely different from the diagnostic signatures in stroma.

In a recent study, a genome-wide LOH/allelic imbalance (AI) scan of DNA was conducted to identify LOH/AI hot/cold spots in prostate epithelium, or in prostate stroma, or in both which identified 156 gene associated with clinicopathologic phenotypes including relapse [14]. Four genes (C7, SLPI, HOXB13, PDCD10) are shared with our 194 stroma prognostic genes with a *p* value of 0.08. Thus, gene expression of a few genes we identified as of potential prognostic value might be altered due to genotypic changes, and are of particular future interest, but most genes we identified do not yet show such an association.

A subset of the more aggressive samples in our study will have reactive stroma, which has been shown to correlate with poor outcome [15]. Thus, we compared the 194 stroma-expressed genes that we found to correlate with outcome to the 1150 genes that were differentially expressed between the “reactive stroma” subgroup of prostate cancer samples and distant stroma from the same 17 patients [15]. Ten genes (RABEP1, ZNF263, MCCC2, SLC4A4, TP53, KPNA6, PTPRF, CDH1, SCNN1A, and CD24) were in common between the studies (*p* value = 0.1312, by a simulation-based test). Another recent study identified 36 prognostic markers also specifically drawn from reactive stroma [16]. In addition, the test samples had substantial tumor present, leaving open the possibility that some genes were differentially expressed between the tumor epithelium of high- and low-risk tumor. Despite these differences in experimental design, four genes (NKX3-1, FOLH1, AGR2, HOXB13) are in common with our 194 stroma prognostic genes with a *p* value of 0.0001, indicating substantial agreement. Moreover, all four gene products are well documented diagnostic or prognostic biomarkers for prostate cancer [31]–[35]. These genes will be of particular interest in future studies.

We analyzed the biological functions for the prognostic 19 probe sets (15 genes) (**Table** 1) using DAVID and MetaCore software. The results indicated that 7 known genes (GADD45B, CDKN1A, NLRP1, ERBB3, YWHAE, TNFSF10 and EIF5A) are related to apoptosis and 6 known genes (CDKN1A, NLRP1, ERBB3, YWHAE, TNFSF10 and EIF5A) are related to cell death, with 6 in common. This is intriguing based on our speculation of tumor-stroma dialog that favors tumor progression. Perhaps, aggressive tumors paracrine signals provide a mechanism to compel the surrounding stroma to undergo remodeling and/or apoptotic processes to facilitate tumor growth and invasion [36] followed by Epithelial-mesenchymal transition [37], [38]. Evidence from independent experiments at the molecular level is needed to support this hypothesis.

We further analyzed the 194 genes (the combination of the 131 probe sets identified by PAM analysis and the 115 probe sets identified from correlation analysis) using a pathway software MetaCore. The result of pathway analysis by using ‘smooth muscle + disease biomarker’ as a filtering parameter indicated that this set of 194 genes are significantly enriched in genes associated with ‘prostatic neoplasms transcription’. The seven genes associated with this description were NCOA3 (TRAM-1), c/EBP (CEBP), NR77 (NR4A1), NK31 (NKX3-1), P53 (TP53), KL5 (KL5), CEBPD. Moreover, 3 genes STAT1, ERBB3, P21 (CDKN1A) were found to be associated with ‘prostatic neoplasms regulation of progression through cell cycle’ and 1 gene STAT1 is associated with ‘prostatic neoplasms inflammatory response’. Pathway analysis using ‘smooth muscle + disease’ as a filtering parameter indicated that 67 of the 194 genes are known to be significantly associated with prostatic diseases and 66 of these 194 genes are known to be significantly associated with prostatic neoplasms (**Table S1**) of which 59 are in common among the two lists. Furthermore, most of these 194 genes are also associated with other cancers such as colorectal neoplasms, breast neoplasms and lung neoplasms, indicating these genes may be commonly involved in cancer related pathways. The pathway analysis also showed that a significant fraction of these 194 genes interact with transcriptional factors, such as P53, SP1, FOXO3A, AR, BCL6, STAT5A, STAT5B, C-Jun, NRF2, MYOD and STAT1, which play crucial roles in cancer development and progression. For example, transcriptional factor SP1 is functionally associated with 94 genes from the 194 gene list (**Figure S4**). SP1 is a transcriptional factor that is over expressed in a variety of cancers and regulates gene expression by interacting with GC rich SP1 binding sites [39]. We also analyzed the 14 common genes (RABEP1, ZNF263, MCCC2, SLC4A4, TP53, KPNA6, PTPRF, CDH1, SCNN1A, CD24, NKX3-1, FOLH1, AGR2, and HOXB13) between the 194 genes and the genes reported in the other two reactive stroma studies [15], [16] using MetaCore, which identified the cell adhesion (cadherin mediated cell adhesion) as the top ranked pathway associated with these overlapped genes. Dysfunction of the cadherin pathways have been reported in various cancers including prostate cancer [40]. The association of prostate cancer and other neoplasms with many genes identified as predominately stroma expressed supports the thesis that the prognostic genes identified here may play functional roles in stroma significantly influence the outcome of prostate cancer.

In summary we conclude that tumor-adjacent prostate cancer stroma contains numerous changes in gene expression at the time of diagnosis that correlate with the chance of relapse following prostatectomy. Moreover, these changes can be harnessed to provide an objective prediction of outcome on an individual basis. It is likely that the differences in RNA expression are often reflected in differences in chromatin modification, DNA methylation, and protein levels, which could also serve as stromal markers for progression.

## Supporting Information

Figure S1 The plot of expression level *vs.* the DFS time for the 19 probe sets from stroma, associated with tumor recurrence. The y axis is the log transformed Affymetrix expression values, x axis is the time to relapse, rho is the Pearson’s correlation coefficient, and the p is the p value for the correlation test.
(PDF)

Figure S2 Heat map of the 227 probe sets (the combination of the 131 differentially expressed probe sets and the 115 DFS associated probe sets) in the 18 training cases. The cases labeled with red are high-risk stroma samples and the cases labeled with green are low-risk stroma samples.
(PDF)

Figure S3 Volcano plot of probe set ratios and probabilities based on 18 training samples.
(PDF)

Figure S4 Among the 194 stroma genes correlated with tumor prognosis there are 94 genes that are functionally associated with transcriptional factor SP1 (p value <1e-6).
(PDF)

Table S1 MetaCore pathway analysis of the 194 genes (the combination of the 131 probe sets identified by PAM analysis and the 115 probe sets identified from correlation analysis) using ‘smooth muscle + disease biomarker’ and ‘prostatic neoplasms transcription’ as filtering parameters.
(XLS)
