# Correction: Expression Changes in the Stroma of Prostate Cancer Predict Subsequent Relapse

**DOI:** 10.1371/annotation/7de63575-e5c9-4f1d-bb45-fc6420e92c71

**Published:** 2012-09-05

**Authors:** Zhenyu Jia, Farah B. Rahmatpanah, Xin Chen, Waldemar Lernhardt, Yipeng Wang, Xiao-Qin Xia, Anne Sawyers, Manuel Sutton, Michael McClelland, Dan Mercola

The authors would like to include more information for one of the listed grants. The grant number for the grant from the Chao Family Comprehensive Cancer Center at University of California of Irvine (ZJ and DAM) is P30CA62203. 

