# Supplementary figures and images for: Expression Changes in the Stroma of Prostate Cancer Predict Subsequent Relapse
Source: PLoS One. 2012 Aug 1;7(8):e41371. doi: 10.1371/journal.pone.0041371 (PMC3411675; doi:10.1371/journal.pone.0041371)

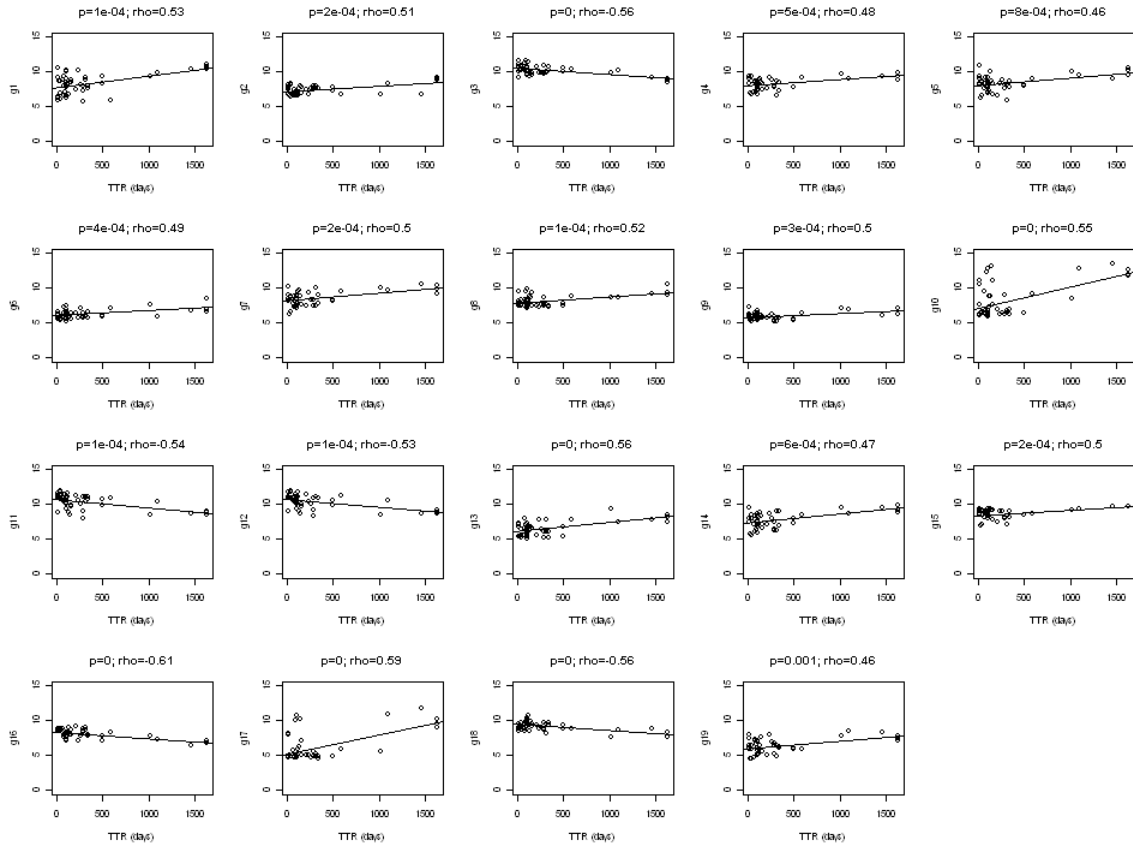

Supplement: Figure S1 — The plot of expression level vs. the DFS time for the 19 probe sets from stroma, associated with tumor recurrence. The y axis is the log transformed Affymetrix expression values, x axis is the time to relapse, rho is the Pearson’s correlation coefficient, and the p is the p value for the correlation test. (PDF) [file pone.0041371.s001.pdf]

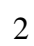

Supplement: Figure S2 — Heat map of the 227 probe sets (the combination of the 131 differentially expressed probe sets and the 115 DFS associated probe sets) in the 18 training cases. The cases labeled with red are high-risk stroma samples and the cases labeled with green are low-risk stroma samples. (PDF) [file pone.0041371.s002.pdf]

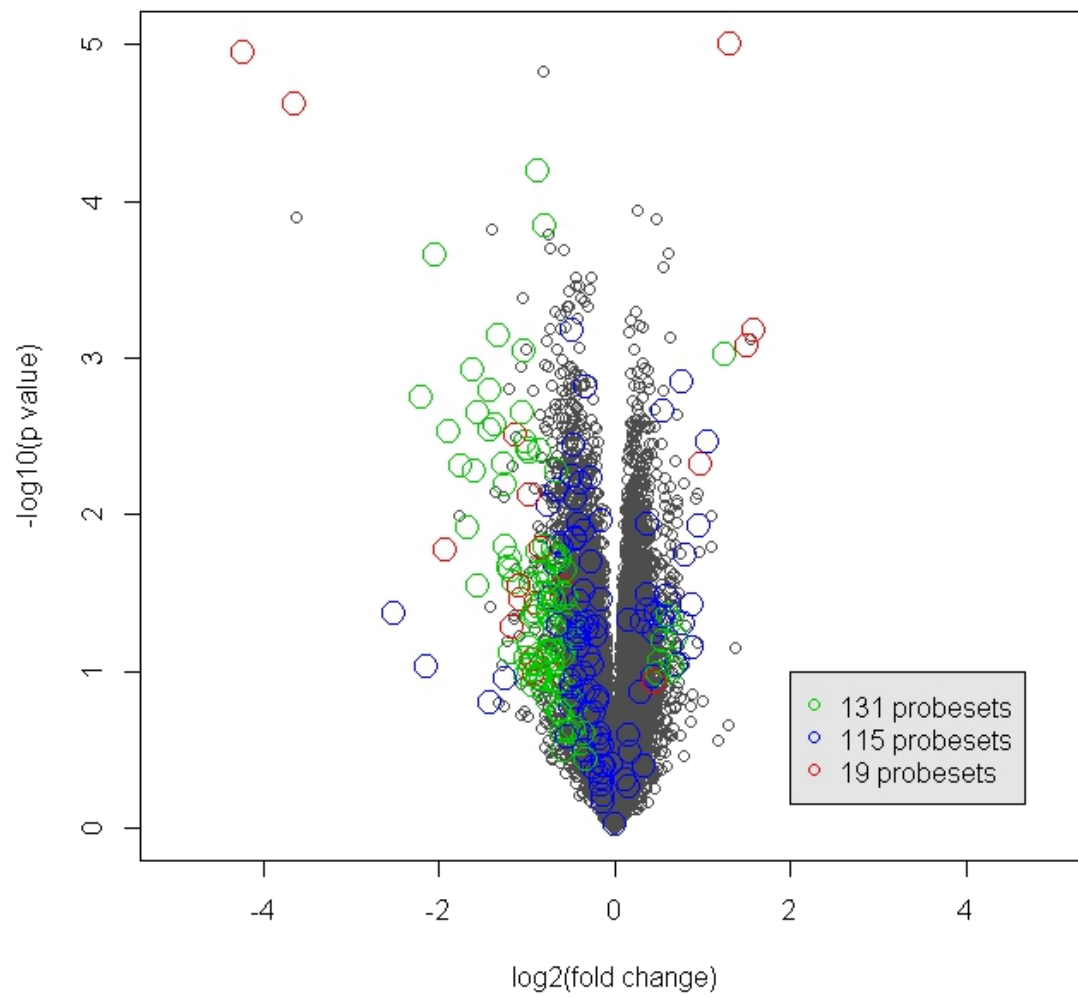

Supplement: Figure S3 — Volcano plot of probe set ratios and probabilities based on 18 training samples. (PDF) [file pone.0041371.s003.pdf]

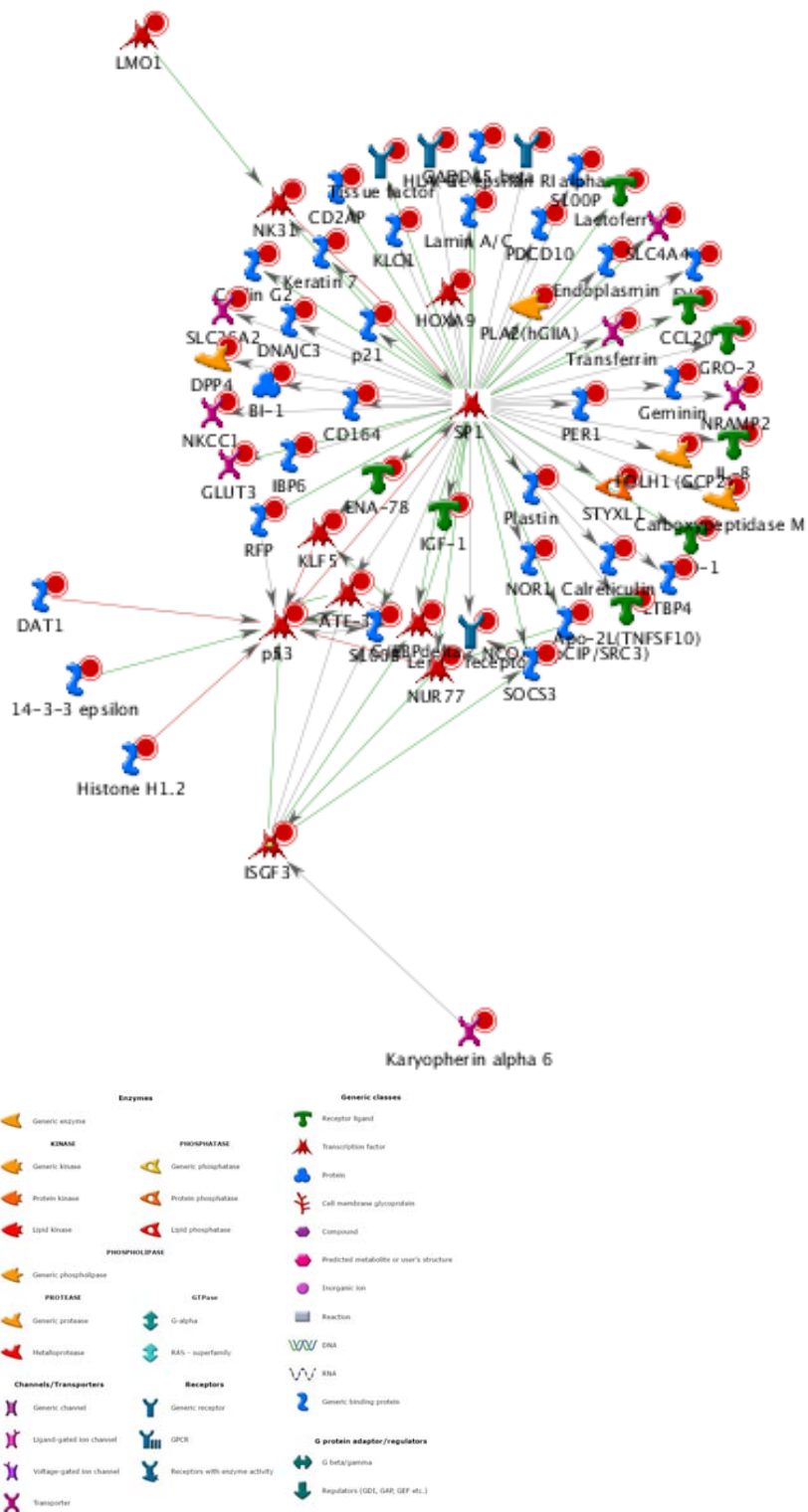

Supplement: Figure S4 — Among the 194 stroma genes correlated with tumor prognosis there are 94 genes that are functionally associated with transcriptional factor SP1 (p value <1e-6). (PDF) [file pone.0041371.s004.pdf]
